# The Diagnostic Accuracy of Metagenomic Next-Generation Sequencing in Diagnosing *Pneumocystis* Pneumonia: A Systemic Review and Meta-analysis

**DOI:** 10.1093/ofid/ofad442

**Published:** 2023-08-18

**Authors:** Aysun Tekin, Hong Hieu Truong, Lucrezia Rovati, Amos Lal, Danielle J Gerberi, Ognjen Gajic, John C O’Horo

**Affiliations:** Division of Nephrology and Hypertension, Department of Internal Medicine, Mayo Clinic, Rochester, Minnesota, USA; Division of Nephrology and Hypertension, Department of Internal Medicine, Mayo Clinic, Rochester, Minnesota, USA; Division of Pulmonary and Critical Care Medicine, Department of Internal Medicine, Mayo Clinic, Rochester, Minnesota, USA; School of Medicine and Surgery, University of Milano-Bicocca, Milan, Italy; Division of Pulmonary and Critical Care Medicine, Department of Internal Medicine, Mayo Clinic, Rochester, Minnesota, USA; Mayo Clinic Library Services, Mayo Clinic College of Medicine and Science, Mayo Clinic, Rochester, Minnesota, USA; Division of Pulmonary and Critical Care Medicine, Department of Internal Medicine, Mayo Clinic, Rochester, Minnesota, USA; Division of Pulmonary and Critical Care Medicine, Department of Internal Medicine, Mayo Clinic, Rochester, Minnesota, USA; Division of Infectious Diseases, Mayo Clinic, Rochester, Minnesota, USA

**Keywords:** diagnostic accuracy, meta-analysis, metagenomic next-generation sequencing, PCP, *Pneumocystis*, systematic review

## Abstract

**Background:**

*Pneumocystis* pneumonia (PCP) is a growing concern as the immunocompromised population expands. Current laboratory approaches are limited. This systematic review aimed to evaluate metagenomic next-generation sequencing (MNGS) tests’ performance in detecting PCP.

**Methods:**

Five databases were searched through December 19, 2022, to identify original studies comparing MNGS with clinically diagnosed PCP. To assess the accuracy, symmetric hierarchical summary receiver operating characteristic models were used.

**Results:**

Eleven observational studies reporting 1442 patients (424 with PCP) were included. Six studies focused exclusively on recipients of biologic immunosuppression (none with HIV-associated immunosuppression). Six were exclusively on bronchoalveolar lavage, while 1 was on blood samples. The sensitivity of MGNS was 0.96 (95% CI, 0.90–0.99), and specificity was 0.96 (95% CI, 0.92–0.98), with negative and positive likelihood ratios of 0.02 (95% CI, 0.01–0.05) and 19.31 (95% CI, 10.26–36.36), respectively. A subgroup analysis of studies exclusively including bronchoalveolar lavage (BAL) and blood samples demonstrated a sensitivity of 0.94 (95% CI, 0.78–0.99) and 0.93 (95% CI, 0.80–0.98) and a specificity of 0.96 (95% CI, 0.88–0.99) and 0.98 (95% CI, 0.76–1.00), respectively. The sensitivity analysis on recipients of biologic immunosuppression showed a sensitivity and specificity of 0.96 (95% CI, 0.90–0.98) and 0.94 (95% CI, 0.84–0.98), respectively. The overall confidence in the estimates was low.

**Conclusions:**

Despite the low certainty of evidence, MNGS detects PCP with high sensitivity and specificity. This also applies to recipients of biologic immunosuppression and tests performed exclusively on blood samples without the need for BAL. Further studies are required in individuals with HIV-associated immunosuppression.

The immunocompromised population is growing as medical technology advances [1]. Opportunistic infections, such as *Pneumocystis* pneumonia (PCP), represent a concern for these individuals, with rising incidence rates [2, 3] and high mortality [4]. Effective treatment for PCP is available, making timely diagnosis the primary determinant of prognosis [5, 6]. Definitive diagnosis depends on microscopic visualization of fungal structures in respiratory samples. These tests, however, have a low sensitivity [1, 6, 7]. Though the polymerase chain reaction (PCR) test was proposed as an alternative, its applicability is limited due to the lack of validation, standardization, and approval in certain countries such as the United States and China [2, 8]. Furthermore, these tests necessitate a presumptive diagnosis of PCP. Given the nonspecific presentation, a presumptive diagnosis of PCP might not be straightforward [1, 2, 5, 8–11]. Additionally, conventional techniques mostly require invasive procedures to obtain an acceptable specimen, which may be impractical in certain conditions [8].

Metagenomic next-generation sequencing (MNGS) has been shown to detect certain infectious agents in a fast, impartial, and hypothesis-free manner [2–4, 12–14]. Furthermore, it has the capacity to identify coinfecting organisms [9, 12, 15]. Moreover, studies have demonstrated a potential of MNGS to identify hematologic dissemination via blood testing, which may obviate the need for invasive procedures [5, 8, 9, 15]. These findings suggest that MNGS might be a promising alternative for diagnosing infections caused by difficult-to-detect organisms like *Pneumocystis jirovecii*.

The clinical significance of positive MNGS findings, however, has been questioned due to the lack of standardization in interpretation [1, 4, 6, 16]. There is a paucity of data comparing MNGS with the gold standard diagnostic methods [13]. Studies of the effect of MNGS on PCP diagnosis have small sample sizes and have mostly been restricted to single centers, limiting their applicability [17]. Thus, we aimed to evaluate and summarize the existing literature on the diagnostic performance of MNGS in detecting PCP and to conduct a meta-analysis of the test's diagnostic performance compared with clinical diagnosis.

## METHODS

The reporting of this study adhered to the Preferred Reporting Items for Systematic Reviews and Meta-Analyses (PRISMA) recommendations [18].

### Search Strategy

A comprehensive search of 5 databases was performed on December 19, 2022. No language, date, or sample size limits were applied. Animal studies were excluded. The literature was searched by a professional medical librarian (D.G.), with input from reviewers (J.C.O., A.T.) for the concepts of metagenomic next-generation sequencing and *Pneumocystis* infections. Search strategies were created using a combination of keywords and standardized index terms. Searches were run in Ovid Cochrane Central Register of Controlled Trials (1991+), Ovid Embase (1974+), Ovid Medline (1946+, including epub ahead of print, in-process, and other nonindexed citations), Scopus (1788+), and Web of Science Core Collection (Science Citation Index Expanded 1975+ and Emerging Sources Citation Index 2015+). After removing conference abstracts and animal studies, a total of 622 citations were retrieved. Deduplication was performed in Covidence and manually, leaving 254 abstracts. Full search strategies are provided in the Supplementary Data.

### Eligibility Criteria

As widely accepted conventional diagnostic methods for *Pneumocystis jirovecii* are highly restricted in terms of sensitivity and specificity, we considered a diagnosis of PCP based on clinical evaluation to be the gold standard reference criterion, regardless of confirmatory conventional laboratory tests. We included original studies that (1) used MNGS on clinical specimens, (2) compared MNGS results with clinically diagnosed or adjudicated PCP diagnosis, and (3) reported information to allow extraction or calculation of the number of patients with true-positive, false-positive, false-negative, and true-negative MNGS results. No restrictions were applied in terms of patient baseline characteristics. Non-peer-reviewed articles were excluded. Studies comparing MNGS with other laboratory tests without clinical confirmation were also excluded. Due to the unavailability of information regarding false positives and true negatives, studies that lacked a patient or sample group not infected with *Pneumocystis jirovecii* were excluded. The corresponding authors of 18 manuscripts that reported on the diagnostic performance of MNGS in detecting pneumonia, not otherwise specified, were contacted to inquire about the availability of the results specifically for PCP diagnosis. They were provided with 12 days to respond, with a reminder email after a week. Five authors (27.8%) responded, 1 of whom provided the necessary data.

At least 2 reviewers independently assessed each study. Any study that did not have a consensus between the 2 reviewers (A.T., H.H.T.) at the abstract and title screen process was included in the full-text review phase. Subsequently, full-text manuscripts were evaluated (A.L., L.R., A.T., H.H.T.) to identify studies that met our criteria for data extraction. Any discrepancies during this stage were addressed between the 2 reviewers to reach a consensus. A third reviewer was consulted in articles where agreement could not be reached after the discussion. The inter-reviewer agreement level at the full-text evaluation phase was evaluated using kappa statistics [19, 20]. The agreement rate was 96%, with a kappa value of 87.5%.

### Data Extraction

The data extraction sheet template was prepared using Microsoft Excel (version 2301) through a pilot trial. The collected variables were those related to the publication (eg, title, publication year), study setting, inclusion and exclusion criteria, baseline characteristics of enrolled patients, methodology for reference method, sample types for MNGS, and test results. If available, the stringently mapped read numbers for the MNGS and the impact of the results on patient management were noted. All manuscripts were extracted by at least 2 reviewers independently (L.R., A.T., H.H.T.). Any discrepancies throughout the extraction step were discussed among the 2 reviewers to reach a consensus.

### Risk of Bias Assessment

Via the Quality Assessment of Diagnostic Accuracy Studies-2 (QUADAS-2) tool, at least 2 reviewers (A.L., L.R., A.T., H.H.T.) independently assessed the methodological quality of the included studies [21]. Reviewers added a question to the risk of bias evaluation for the reference standard section (ie, Was the protocol specified for reference clinical evaluation?). Disagreements between reviewers were addressed by including a third reviewer's evaluation as a tiebreaker.

### Author Contact

After the extraction of the study data and evaluation of the risk of bias, the collected information for each study was shared with the corresponding authors for their confirmation. If no reply was received by the end of the 1-week deadline, originally collected data were used.

### Outcomes

Diagnostic odds ratio (DOR), sensitivity, specificity, positive likelihood ratio (PLR), and negative likelihood ratio (NLR) were calculated and reported along with their 95% confidence intervals to summarize the diagnostic accuracy of MNGS in detecting PCP.

### Synthesis of Results

True-positives, true-negatives, false-positives, and false-negatives of the MNGS were extracted or calculated from the metrics provided in the studies. Using these values, sensitivity and specificity, NLR, PLR, and DOR were calculated to evaluate the performance of MNGS. Our reference diagnostic method, PCP diagnosis based on clinical evaluation, is imperfect and prone to heterogeneity due to the varying definitions accepted by the study teams as well as the subjective evaluations. To address this and to account for the correlation between sensitivity and specificity, we used hierarchical summary receiver operating characteristics (HSROC) combined with a diagnostic random-effects model (with the Sidik Jonkman method) to generate pooled estimates [22, 23]. Also, HSROC curves were drawn based on the estimates. Potential publication bias was evaluated by the review of studies that were industry-supported and abstracts that were not published as manuscripts.

Heterogeneity was analyzed by visual apprising the forest plots for NLR and PLR [24]. To explore heterogeneity, we predetermined the potential sources and planned subgroup analyses according to sample type (ie, blood vs bronchoalveolar lavage [BAL]), presence of immunosuppression, and risk of bias (ie, high vs low). Though there are multiple domains of the QUADAS-2 tool in terms of evaluating the risk of bias, we prespecified our domain of interest in terms of the subgroup analysis as the reference standard risk of bias. That is because a major factor impacting that domain was the blinding of the reference method (ie, clinical diagnosis) to MNGS results, which we felt was the most impactful. We also conducted a sensitivity analysis based on the funding source, performing the calculations solely on studies that were not industry sponsored. A subgroup analysis based on the type of immunosuppression (ie, HIV-associated vs non-HIV-associated) was planned but never carried out due to the lack of studies reporting on patients with HIV-associated immunosuppression.

Statistical analysis was performed on OpenMeta[Analyst] software [25].

### Grading the Quality of Evidence

Overall confidence in the estimates was assessed following the Grading of Recommendation, Assessment, Development, and Evaluation (GRADE) approach recommended for diagnostic accuracy studies [24]. Regarding the diagnostic nature of the included observational studies, we started with high certainty and rated it down according to the evaluation of methodological quality based on the suggested domains. As heterogeneity is expected to be high in diagnostic accuracy studies, it was not factored in while determining the certainty in estimates [26].

## RESULTS

**Figure 1.**
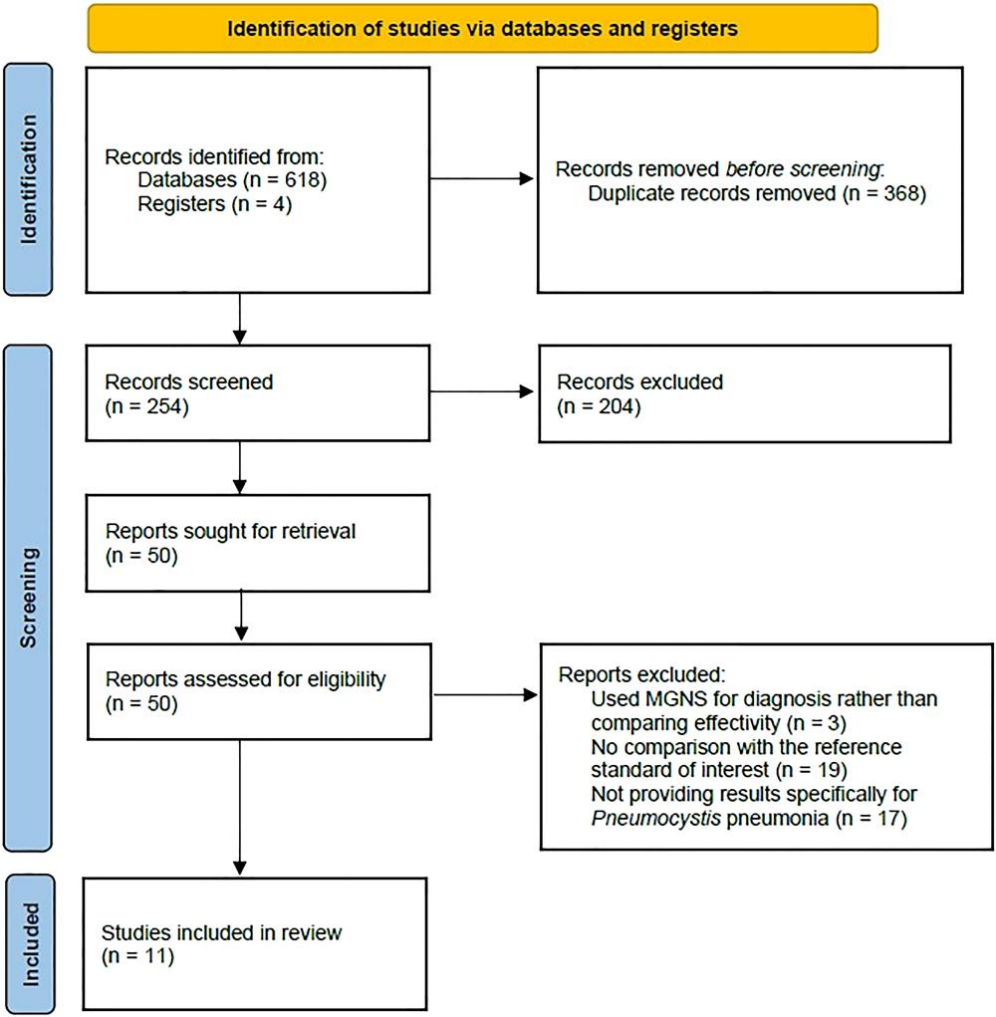
Flowchart for the identification of studies [18]. Abbreviation: MNGS, metagenomic next-generation sequencing.

Following the evaluation of abstracts and manuscripts retrieved by the search, 11 studies were included in the final analysis (Figure 1) [5, 8, 17, 27–34].

The sample size of the studies ranged from 53 to 310 patients, with a median (interquartile range) of 72 (60–194). The proportion of PCP patients ranged from 0.6% to 64.6%. In total, studies included 1442 patients with a pooled PCP frequency of 29.4% (n = 424). Three studies did not report any specific underlying condition, while 6 were exclusively conducted on recipients of biologic immunosuppression. One of the latter studies specifically focused on renal comorbidities, while 2 others enrolled solely patients with rheumatological conditions. Among the reported causes of immunosuppression, connective tissue disorders were the most common (n = 333, 38.9%), followed by solid organ transplant history and solid tumors (n = 159 each, 19.7%). None of the studies enrolled patients with HIV-associated immunosuppression. Characteristics of included studies are reported in Table 1. Only 3 studies reported the time to diagnosis for MNGS, with 1 reporting an average (SD) of 30.54 (10.32) hours and the others reporting 24 and 24–48 hours.

**Table 1.** Study Characteristics

Author, Publication Year	Country	Patient Enrollment Period	Setting	Study Type	Immunocompromised Patients^a^	Sample Type	TP	TN	FP	FN
Gaston et al. [27]	USA	December 2019–January 2021	Multicenter	Cohort	No	Exclusively BAL	0	175	1	1
Gu et al. [28]	China	August 2018–December 2019	Single center	Case control	Yes	Exclusively blood	35	25	0	2
Jiang et al. [5]	China	July 2018–November 2020	Single center	Case control	No	Respiratory and blood	60	129	5	0
Li, Jia et al. [8]	China	January 2019–January 2021	Single center	Cohort	Yes	Respiratory and blood	18	54	0	0
Li, Jun et al. [29]	China	May 2019–June 2021	Single center	Cohort	No	Respiratory, blood, and tissue	11	42	0	0
Lin et al. [30]	China	September 2018–December 2020	Single center	Cohort	Yes	Exclusively BAL	26	37	6	0
Peng et al. [31]	China	April 2017–April 2019	Single center	Cohort	Yes	Exclusively BAL	30	29	1	0
Shi et al. [32]	China	January 2019–December 2020	Single center	Cohort	Yes	Exclusively BAL	22	34	0	2
Sun et al. [33]	China	May 2017–May 2021	Multicenter	Cohort	Yes	Exclusively BAL	75	103	18	2
Wang, Chengtan et al. 2022	China	August 2020–February 2022	Multicenter	Cohort	No	Exclusively BAL	18	288	4	0
Wang, Dongsheng et al. [17]	China	January 2019–January 2022	Single center	Cohort	No	Respiratory and blood	122	61	6	0

Abbreviations: BAL, bronchoalveolar lavage; FN, false negative; FP, false positive; TN, true negative; TP, true positive.

a Did the study exclusively include recipients of biologic immunosuppression?

### Quality Assessment of Included Studies and Evaluation of the Certainty of Evidence

**Figure 2.**
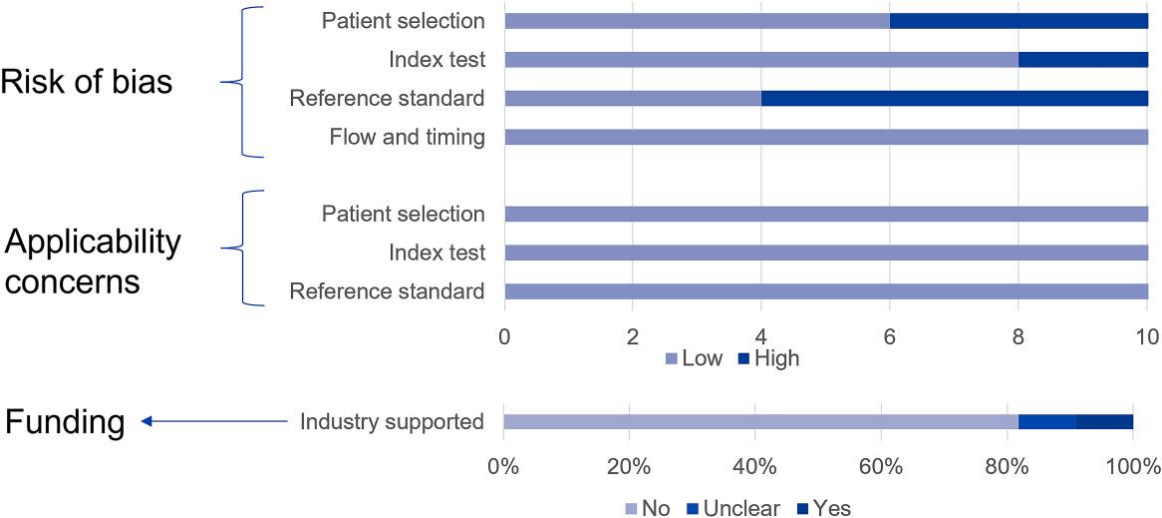
Summary of quality assessment of studies using the Quality Assessment of Diagnostic Accuracy Studies-2 tool [21].

The reference standard domain had the highest frequency of high risk of bias in the included studies (n = 7, 63.6%), owing to the reference standard not being blind to the MNGS results. All included studies provided a detailed approach, specifying their methodology for the reference standard. There were no major concerns about the applicability of the studies or the flow and timing. The quality assessment evaluation of the studies via the QUADAS-2 tool is summarized in Figure 2, and the detailed evaluation results are provided in Supplementary Table 1.

As for the funding resources of the included studies, 1 received industry support, and 1 did not specify a funding source. The remaining studies were funded by national grants or institutions (Figure 2).

All included studies were observational studies evaluating the performance of MNGS as a case–control approach or cohort study, comparing the results with an appropriate reference standard. Though 3 out of 11 studies mentioned the potential impact of the test results on patient follow-up, there was no detailed information about the potential influence of the test on patient-important outcomes, introducing indirectness. Although the confidence intervals for sensitivity, specificity, and likelihood ratios were relatively high, they did not cross a clinical decision threshold, and we decided not to downgrade the confidence in the estimates. In terms of publication bias assessment, the majority of the studies were not industry-funded, and we only encountered 1 abstract that was not published as a complete manuscript out of the 50 publications evaluated in full-text review (2%). Thus, we concluded that publication bias was not a major concern. However, as mentioned in the quality assessment, there was a high risk of bias in the included studies. Accordingly, we concluded that the evidence gathered in this meta-analysis warrants low confidence.

### Diagnostic Accuracy of MNGS for PCP

**Figure 3.**
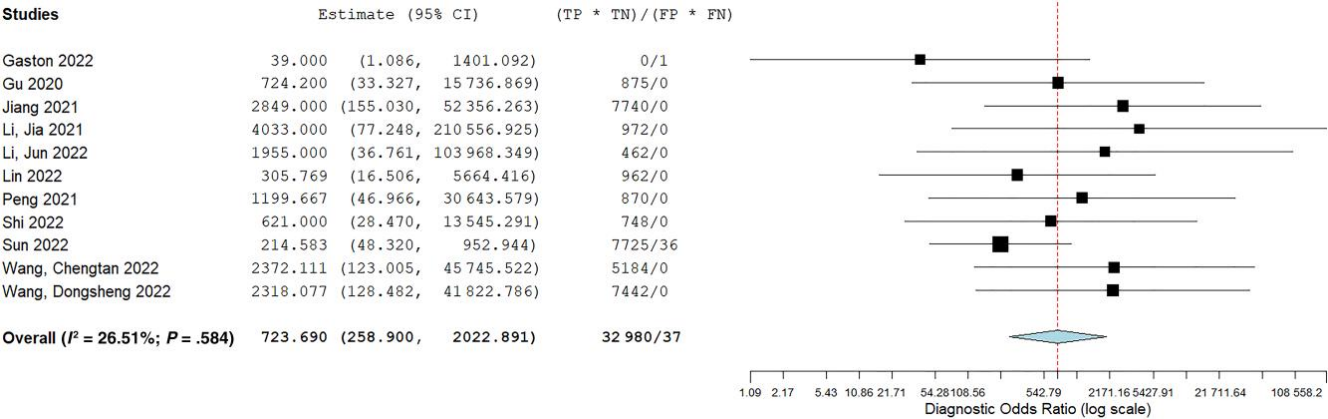
Summary forest plot for diagnostic odds ratios of all included studies.

**Figure 4.**
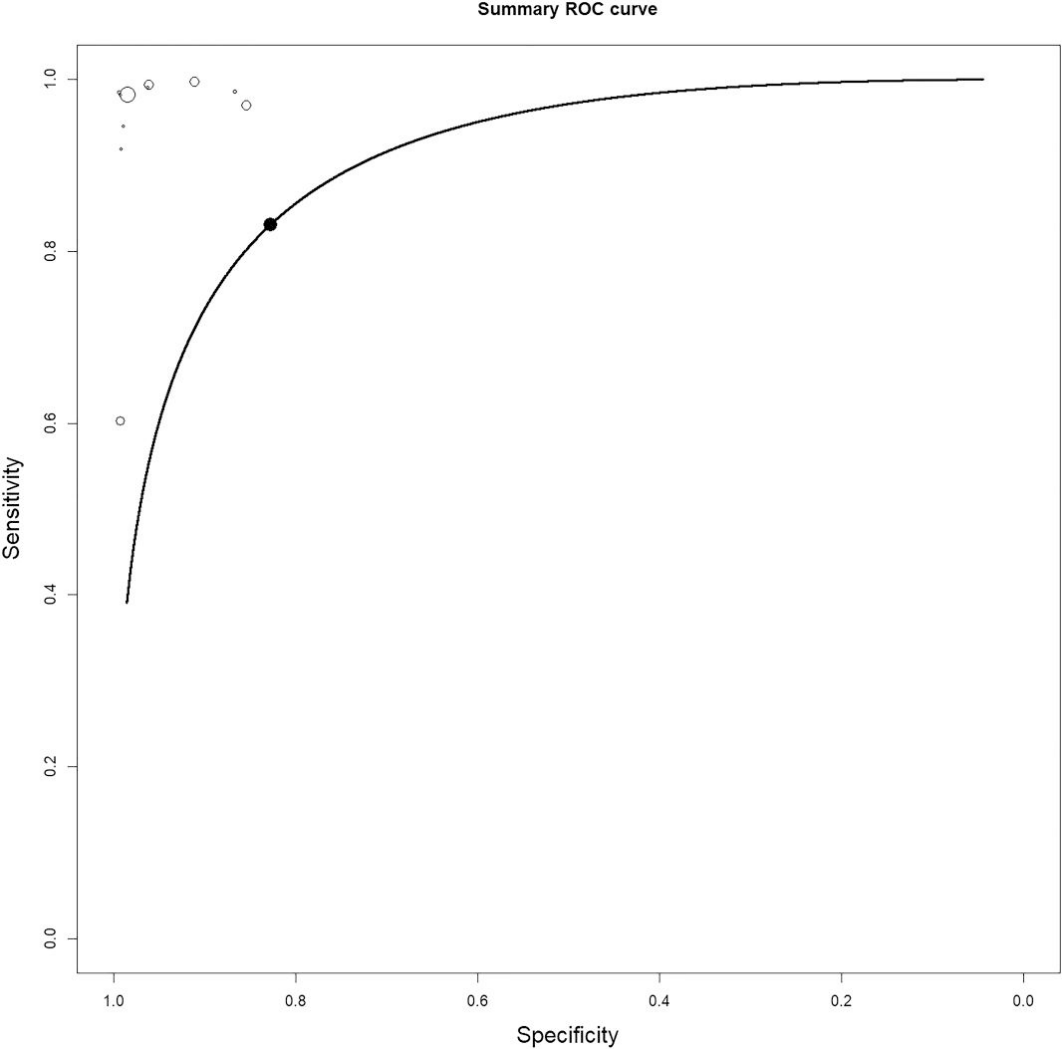
Summary receiver operating characteristic curves of all included studies. Abbreviation: ROC, receiver operating characteristics.

Among the 11 studies, the sensitivity ranged from 0.25 (95% CI, 0.013–0.891) to 0.996 (95% CI, 0.938–1.0), while the specificity ranged from 0.851 (95% CI, 0.776–0.904) to 0.992 (95% CI, 0.959–0.998). In the pooled analyses, the sensitivity of MGNS was 0.96 (95% CI, 0.90–0.99), and specificity was 0.96 (95% CI, 0.92–0.98). Forest plots for pooled results for sensitivity and specificity are shown in Supplementary Figure 1a and b. They are also outlined in Table 2, along with other parameters. The diagnostic odds ratio of MNGS compared with clinically diagnosed PCP was 723.69 (95% CI, 258.9–2022.9) (Figure 3). The receiver operating characteristic curve is shown in Figure 4. Heterogeneity was high in both the NLR and PLR analyses, possibly attributable to the different sample types, patient characteristics, or the risk of bias.

**Table 2.** Summary of Diagnostic Accuracy for Different Subgroups and the Sensitivity Analyses

Analysis	Patient Group	No. of Studies	No. of Patients, PCP/Total	Diagnostic Odds Ratio (95% CI)	Sensitivity (95% CI)	Specificity (95% CI)	Negative Likelihood Ratio (95% CI)	Positive Likelihood Ratio (95% CI)
All	All included patients	11	424/1442	723.69 (258.9–2022.9)	0.96 (0.90–0.99)	0.96 (0.92–0.98)	0.02 (0.01–0.05)	19.31 (10.26–36.36)
BAL vs Blood	Exclusively BAL	6	176/872	412.07 (124.66–1362.09)	0.94 (0.78–0.99)	0.96 (0.88–0.99)	0.04 (0.01–0.1)	16.57 (6.87–39.97)
	Exclusively blood	1	37/62	724.2 (33.33–15736.87)	0.93 (0.80–0.98)	0.98 (0.76–1.00)	0.07 (0.00–1.05)	48.58 (3.12–757.11)
Risk of bias	High	7	232/916	753.61 (187.99–3021)	0.96 (0.82–0.99)	0.96 (0.91–0.99)	0.03 (0.01–0.09)	20.45 (9.23–45.34)
	Low	4	192/526	695.33 (218.21–1910.21)	0.97 (0.91–0.99)	0.95 (0.84–0.99)	0.02 (0.01–0.05)	18.87 (5.92–60.14)
Sensitivity analysis	Exclusively recipients of biologic immunosuppression	6	212/519	469.99 (145.73–1515.8)	0.96 (0.90–0.98)	0.94 (0.84–0.98)	0.03 (0.02–0.05)	15.02 (5.66–39.84)
Sensitivity analysis	No industry funding	10	423/1265	819.77 (312.87–2147.9)	0.97 (0.93–0.99)	0.95 (0.90–0.98)	0.02 (0.01–0.03)	19.4 (9.89–38.11)

Abbreviations: BAL, bronchoalveolar lavage; PCP, *Pneumocystis* pneumonia.

### Subgroup and Sensitivity Analyses

The summary results for subgroup and sensitivity analyses are shown in Table 2, and the forest plots for DOR are provided in Supplementary Figure 2.

In the subgroup analyses, the test's diagnostic accuracy was evaluated separately for BAL and blood samples, and it was demonstrated that the test effectively detected the condition in both types of samples (sensitivity, 0.94; 95% CI, 0.78–0.99; and sensitivity, 0.93; 95% CI, 0.80–0.98; specificity, 0.96; 95% CI, 0.88–0.99; and specificity, 0.98; 95% CI, 0.76–1.00; for BAL and blood samples, respectively). The overall heterogeneity of the likelihood ratios remained high.

In the subgroup analysis comparing the studies with high and low risk of bias according to the reference standard domain of the QUADAS-2 tool, sensitivity and specificity were high in both groups (sensitivity, 0.96; 95% CI, 0.82–0.99; and sensitivity, 0.97; 95% CI, 0.91–0.99; specificity, 0.96; 95% CI, 0.91–0.99; and specificity, 0.95; 95% CI, 0.84–0.99; for high- and low-risk studies, respectively). The heterogeneity was still considerable among both groups.

When a sensitivity analysis was conducted on studies focusing exclusively on recipients of biologic immunosuppression, the test still had an acceptable diagnostic accuracy, with sensitivity and specificity levels of 0.96 (95% CI, 0.90–0.98) and 0.94 (95% CI, 0.84–0.98), respectively. Heterogeneity was reduced in this analysis visually. The sensitivity analysis, which excluded the industry-sponsored study, also revealed high sensitivity and specificity (sensitivity, 0.97; 95% CI, 0.93–0.99; and specificity, 0.95; 95% CI, 0.90–0.98), with similar heterogeneity to the general analysis.

The remaining heterogeneity issues in subgroup and sensitivity analyses were thought to be due to factors that were not addressed in these analyses, such as differences in the index test application or interpretation, methodology for the reference diagnosis, or the varying prevalence of PCP in patient samples.

## DISCUSSION

We conducted a systematic review assessing the performance of MNGS in diagnosing PCP. Due to the limitations of the currently available tests, there is a need for a high-performance diagnostic method. Eleven published studies comparing MNGS with clinically diagnosed PCP were identified. Evaluation based on the QUADAS-2 tool indicated a high risk of bias either in the patient selection or reference standard domains in all studies. Only 2 of the 11 studies explicitly stated that the reference test, that is, clinical diagnosis, was blinded to MNGS results, which raised concerns about bias. With the exception of 1 study, all studies included a protocol for the clinical evaluation that served as the reference. However, the clinical diagnosis still leaves room for subjectivity, which may have introduced a certain level of heterogeneity and complicated pooling the results. Keeping these factors in mind, the pooled analyses of the diagnostic performance demonstrated that MNGS had good diagnostic accuracy, as evidenced by high DOR, sensitivity, and specificity [35]. This performance was maintained in the subgroup analyses of studies on BAL and blood samples. The findings were also consistent when the analysis was limited to recipients of biologic immunosuppression.

Cell-free next-generation sequencing has been studied in various fields [13, 16, 36]. Though its application to infectious disease diagnostics is relatively new, its diagnostic accuracy has been evaluated in infections across different spectrums, demonstrating levels of sensitivity and specificity ranging from 73.8% to 93% and 68% to 100%, respectively [14, 37–41]. Unlike other diagnostic methods, MNGS provides a hypothesis-free approach to pathogen detection [2, 3, 12]. The rapid turnaround time and capacity to identify coinfecting organisms, even under effective treatment, make MNGS a promising tool for infectious disease diagnostics [9, 12, 15]. It has also been suggested that MNGS might be useful in quantitatively following up the pathogen load to determine treatment response [12]. Nonetheless, its performance is known to be impacted by certain characteristics [42].

Diagnosis of PCP is challenging, partially because *Pneumocystis jirovecii* cannot be grown in vitro and definitive diagnosis requires microscopic visualization [11]. This technique is not widely available and has low sensitivity [4, 6]. In 2020, a comprehensive literature review showed that microscopy techniques on BAL samples without immunofluorescence have >90% negative and positive predictive values with a wide range of sensitivity, as low as 31%. The immunofluorescent staining methods were concluded to have 48%–100% sensitivity and 82%–100% specificity [43]. Serum beta-D-glucan and lactate dehydrogenase tests have been suggested as diagnostic methods. However, there are no clearly identified thresholds, and their use for PCP diagnosis is controversial [4, 17]. In a systematic review and meta-analysis including 23 studies, the pooled sensitivity of the serum beta-D-glucan test in diagnosing PCP was 91% with a specificity of 79%, with a lower diagnostic accuracy in the non–HIV patient group [44]. Although PCR tests have emerged as a rapid and reliable alternative, they are not universally approved for clinical use, and their performance relies on a presumptive diagnosis [2, 4]. Furthermore, distinguishing colonization from infection is difficult using PCR [6, 11]. In the published literature, *Pneumocystis* PCR is reported to have the best sensitivity and specificity, with values ranging from 96% to 98% and 94% to 99%, respectively [45–47]. A direct comparison of MNGS with these tests was outside the scope of this study. Nevertheless, our findings suggest that MNGS has a diagnostic performance comparable to *Pneumocystis* PCR and appears to be more effective than the serum beta-D-glucan test and microscopy methods in diagnosing PCP.

Another potential advantage of MNGS is the potential for detecting *Pneumocystis jirovecii* in blood, obviating the need for invasive procedures, which is especially important in the vulnerable population of immunocompromised patients. Three of the studies included in this meta-analysis reported separate results for blood test results, and all concluded that blood samples provided reliable results [5, 8, 28]. It was also suggested that positive results in blood samples might have a higher specificity for invasive infection rather than potential colonization [5, 9]. Our subgroup analysis showed similarly successful performance on blood and BAL samples.

Our study has many strengths. We had specific study selection criteria, which were a priori defined by our study protocol. In the absence of a widely accepted gold standard, we evaluated accuracy by taking PCP diagnosed on clinical grounds into account. We consider this a strength of our study as this is the closest alternative to a gold standard diagnostic method. Furthermore, our study employed a comprehensive literature search, reviewing multiple databases, reproducible evaluations, standardized data collection, and a predefined protocol for subgroup and sensitivity analyses.

Our findings, however, must be interpreted within the context of the study's limitations. First, the reference standard of clinical diagnosis may be flawed due to its subjective nature. Though the inherent limitations of diagnosis pose a challenge for this review, we believe that this work, providing information about alternative and potentially improved methodologies for PCP diagnosis, is important as the new advanced diagnostic practices would help increase the understanding of this disease, ultimately leading to a more streamlined diagnostic process. The potential heterogeneity in definitions and the lack of an aligned definition supported by guidelines [48] are other important limitations of this review. Another shortcoming was the absence of negative controls in the original studies, complicating the interpretation of false-positive results. Furthermore, the certainty in estimates was low according to GRADE criteria for diagnostic studies [24]. All the included studies were observational, with a high risk of bias. Heterogeneity persisted in the subgroup and sensitivity analyses, possibly due to the subjective nature of clinical judgments, inconsistent use of conventional tests, and varying proportions of PCP cases among studies. Another potential contributor to heterogeneity was the lack of standardization in the technique and interpretation of MNGS across the included studies, which we were unable to control. Additionally, specifics regarding the timing of the test (ie, early or late during the disease process) were not reported, preventing us from safely excluding a potential spectrum bias. All studies except 1 were conducted in the same country, raising concerns about the generalizability of the results. Finally, no individuals with HIV-associated immunosuppression were included, limiting the applicability of our findings to this population.

### Implications for Practice and Further Research

For the reliable diagnosis of PCP, MNGS appears to be a promising option. However, further studies with more robust methodologies to reduce the risk of bias are required to produce higher-quality evidence. Only 1 study evaluated the performance of MNGS exclusively in blood samples, which underscores the importance of further investigations to make a more robust recommendation on the utility of MNGS in blood samples. Furthermore, there was a paucity of studies reporting on the impact of MNGS on clinical decision-making, patient management, and patient-important outcomes. Also, very few studies reported the potential impact of MNGS on expediting the diagnostic workup. Though MNGS tests are known to be high-cost, they could be time- and life-saving in certain clinical settings [9]. Thus, more studies evaluating patient-important outcomes and the potential impact on the timeline are necessary to assess cost-effectiveness. Also, quantitative test results, that is, stringent mapped read numbers, were reported in very few studies, emphasizing the need for further studies to potentially standardize the interpretation. Finally, studies are needed to assess the diagnostic accuracy of MNGS in patients with HIV infection.

## CONCLUSIONS

Our analysis demonstrated that MNGS is helpful in detecting PCP with high accuracy, sensitivity, and specificity. This statement holds true for the subgroups of recipients of biologic immunosuppression as well as tests performed on blood samples. Though the certainty of this evidence is low and the cost-effectiveness is unknown at this time, it might be a promising approach for this challenging diagnosis.

## Supplementary Material

ofad442_Supplementary_Data

## Supplementary Data

Supplementary materials are available at *Open Forum Infectious Diseases* online. Consisting of data provided by the authors to benefit the reader, the posted materials are not copyedited and are the sole responsibility of the authors, so questions or comments should be addressed to the corresponding author.

## Notes

***Author contributions.*** A.T.: literature search, data collection, study design, analysis of data, manuscript preparation. H.H.T.: literature search, data collection, study design, analysis of data, manuscript preparation. L.R.: literature search, data collection, study design, analysis of data. A.L.: literature search, data collection, study design. D.J.G.: literature search, data collection. O.G.: study design, review of manuscript. J.C.O.: study design, analysis of data, review of manuscript.

***Data availability.*** The data that support the findings of this study are available from the corresponding author, A.T., upon reasonable request.

***Financial support*.** There are no financial disclosures relevant to this article.

***Patient consent.*** The conduct of this meta-analysis did not necessitate ethics approval or patient consent, as it involved the analysis of existing published data.
