# Supplementary material for: The Diagnostic Accuracy of Metagenomic Next-Generation Sequencing in Diagnosing Pneumocystis Pneumonia: A Systemic Review and Meta-analysis
Source: Open Forum Infect Dis. 2023 Aug 18;10(9):ofad442. doi: 10.1093/ofid/ofad442 (PMC10478158; doi:10.1093/ofid/ofad442)
Supplement: ofad442_Supplementary_Data [file ofad442_supplementary_data.zip › Draft_5_3_supp_material_revision_clean.docx]

**Supplemental Material – Search Strategy:**

**Cochrane Central Register of Controlled Trials (CCTR) via Ovid** (1991+)):

| **#** | **Query** | **Results** |
| --- | --- | --- |
| 1 | (metagenomic* or meta-genomic* or population-genomic* or community-genomic* or mNGS or NGS or ((high-throughput or next-generation or next-gen or modern-nucleic-acid or shotgun or Nanopore or chromatin-immunoprecipitation or massively-parallel or mate-pair or paired-end or hybridization or ligation or SMRT or Chromatin-Immunoprecipitation or deep or ion or cell-free or mcfDNA or illumina) adj2 (sequenc* or resequenc*)) or pyrosequenc* or RNA-Seq or Karius or fluid-biops* or liquid-biops*).ab,hw,ti. | 3,709 |
| 2 | (pneumocysti* or p-carinii or p-jirovecii or PCP-pneumoni* or PCP-infection* or PJP-pneumoni* or PJP-infection* or plasma-cell-pneumoni* or pneumocystos* or jirovecii-pneumoni* or carinii-pneumoni*).ab,ti,hw. | 710 |
| 3 | 1 and 2 | 4 |

**Embase via Ovid** (1974+):

| **#** | **Query** | **Results** |
| --- | --- | --- |
| 1 | metagenomics/ or exp high throughput sequencing/ or liquid biopsy/ | 132,516 |
| 2 | (metagenomic* or meta-genomic* or population-genomic* or community-genomic* or mNGS or NGS or ((high-throughput or next-generation or next-gen or modern-nucleic-acid or shotgun or Nanopore or chromatin-immunoprecipitation or massively-parallel or mate-pair or paired-end or hybridization or ligation or SMRT or Chromatin-Immunoprecipitation or deep or ion or cell-free or mcfDNA or illumina) adj2 (sequenc* or resequenc*)) or pyrosequenc* or RNA-Seq or Karius or fluid-biops* or liquid-biops*).ab,kf,ti,dq. | 255,067 |
| 3 | or/1-2 | 293,723 |
| 4 | exp pneumocystosis/ | 19,084 |
| 5 | (pneumocysti* or p-carinii or p-jirovecii or PCP-pneumoni* or PCP-infection* or PJP-pneumoni* or PJP-infection* or plasma-cell-pneumoni* or pneumocystos* or jirovecii-pneumoni* or carinii-pneumoni*).ab,kf,ti,dq,hw. | 27,891 |
| 6 | or/4-5 | 27,891 |
| 7 | (exp animal/ or animal experiment/ or nonhuman/) not (exp human/ or human experiment/) | 7,054,154 |
| 8 | in vitro study/ or in-vitro.ab,kf,ti,dq. | 2,313,666 |
| 9 | or/7-8 | 8,400,340 |
| 10 | (3 and 6) not 9 | 226 |
| 11 | limit 10 to conference abstract | 36 |
| 12 | 10 not 11 | 190 |

**MEDLINE via Ovid** (1946+ and Epub Ahead of Print, In-Process & Other Non-Indexed Citations and Ovid MEDLINE(R) Daily):

| **#** | **Query** | **Results** |
| --- | --- | --- |
| 1 | Metagenomics/ or exp High-Throughput Nucleotide Sequencing/ or Liquid Biopsy/ | 60,189 |
| 2 | (metagenomic* or meta-genomic* or population-genomic* or community-genomic* or mNGS or NGS or ((high-throughput or next-generation or next-gen or modern-nucleic-acid or shotgun or nanopore or chromatin-immunoprecipitation or massively-parallel or mate-pair or paired-end or hybridization or ligation or SMRT or Chromatin-Immunoprecipitation or deep or ion or cell-free or mcfDNA or illumina) adj2 (sequenc* or resequenc*)) or pyrosequenc* or RNA-Seq or Karius or fluid-biops* or liquid-biops*).ab,kf,ti. | 180,694 |
| 3 | or/1-2 | 196,371 |
| 4 | exp Pneumocystis Infections/ | 9,683 |
| 5 | (pneumocysti* or p-carinii or p-jirovecii or PCP-pneumoni* or PCP-infection* or PJP-pneumoni* or PJP-infection* or plasma-cell-pneumoni* or pneumocystos* or jirovecii-pneumoni* or carinii-pneumoni*).ab,kf,ti,hw. | 14,791 |
| 6 | or/4-5 | 14,791 |
| 7 | exp animals/ not humans/ | 5,074,784 |
| 8 | In Vitro Techniques/ or in-vitro.ab,kf,ti. | 1,662,761 |
| 9 | or/7-8 | 6,104,594 |
| 10 | (3 and 6) not 9 | 98 |

**Scopus via Elsevier** (1788+):

TITLE-ABS-KEY ( metagenomic*  OR  meta-genomic*  OR  population-genomic*  OR  community-genomic*  OR  mngs  OR  ngs  OR  ( ( high-throughput  OR  next-generation  OR  next-gen  OR  modern-nucleic-acid  OR  shotgun  OR  nanopore  OR  chromatin-immunoprecipitation  OR  massively-parallel  OR  mate-pair  OR  paired-end  OR  hybridization  OR  ligation  OR  smrt  OR  chromatin-immunoprecipitation  OR  deep  OR  ion  OR  cell-free  OR  mcfdna  OR  illumina )  W/2  ( sequenc*  OR  resequenc* ) )  OR  pyrosequenc*  OR  rna-seq  OR  karius  OR  fluid-biops*  OR  liquid-biops* )  AND  TITLE-ABS-KEY ( pneumocysti*  OR  p-carinii  OR  p-jirovecii  OR  pcp-pneumoni*  OR  pcp-infection*  OR  pjp-pneumoni*  OR  pjp-infection*  OR  plasma-cell-pneumoni*  OR  pneumocystos*  OR  jirovecii-pneumoni*  OR  carinii-pneumoni* )

**Web of Science Core Collection via Clarivate Analytics** (Science Citation Index Expanded 1975+ & Emerging Sources Citation Index 2015+):

| #3 | #2 AND #1 |
| --- | --- |
| #2 | pneumocysti* or p-carinii or p-jirovecii or PCP-pneumoni* or PCP-infection* or PJP-pneumoni* or PJP-infection* or plasma-cell-pneumoni* or pneumocystos* or jirovecii-pneumoni* or carinii-pneumoni* (Topic) |
| #1 | metagenomic* or meta-genomic* or population-genomic* or community-genomic* or mNGS or NGS (Topic) or pyrosequenc* or RNA-Seq or Karius or fluid-biops* or liquid-biops* (Topic) or ((high-throughput or next-generation or next-gen or modern-nucleic-acid or shotgun or Nanopore or chromatin-immunoprecipitation or massively-parallel or mate-pair or paired-end or hybridization or ligation or SMRT or Chromatin-Immunoprecipitation or deep or ion or cell-free or mcfDNA or illumina) NEAR/2 (sequenc* or resequenc*)) (Topic) |

**Supplemental Table. Risk of bias evaluation with the signalling questions.**

| Author, publication year | Risk of bias | | | | | | | | | | | | | | | | | Applicability | | |
| --- | --- | --- | --- | --- | --- | --- | --- | --- | --- | --- | --- | --- | --- | --- | --- | --- | --- | --- | --- | --- |
|  | **Patient selection** | | | | **Index test** | | | | **Reference standard** | | | | **Flow and timing** | | | | | **^*^** | **^**^** | **^***^** |
|  | **1** | **2** | **3** | **4** | **5** | **6** | **7** | **8** | | **9** | **10** | **11** | **12** | **13** | **14** | **15** | **16** | **17** | **18** | **19** |
| Gaston *et al.*, 2022[27] | Yes | Yes | Unclear | Low | No | Yes | Low | Yes | | Yes | No | High | Yes | Yes | No | Yes | Low | Low | Low | Low |
| Gu *et al*., 2020[28] | Unclear | No | Unclear | High | Unclear | No | High | Yes | | Yes | Yes | Low | Yes | Yes | Yes | Unclear | Low | Low | Low | Low |
| Jiang *et al*., 2021[29] | Yes | No | Yes | High | Unclear | Yes | Low | Yes | | Yes | Unclear | Low | Yes | Yes | Yes | Yes | Low | Low | Low | Low |
| Li, Jia *et al.*, 2021[8] | Unclear | Yes | Unclear | High | Unclear | No | High | Yes | | Yes | Unclear | Low | Yes | Yes | Yes | Unclear | Low | Low | Low | Low |
| Li, Jun *et al.*, 2022[31] | Yes | Yes | Unclear | Low | Yes | Yes | Low | Yes | | Yes | No | High | No | Yes | Yes | Yes | Low | Low | Low | Low |
| Lin *et al.*, 2022[32] | Yes | Yes | Unclear | Low | Yes | Yes | Low | Yes | | Yes | No | High | Unclear | Yes | Yes | Yes | Low | Low | Low | Low |
| Peng *et al.*, 2021[33] | Yes | Yes | Yes | Low | Yes | Yes | Low | Yes | | Yes | No | High | Unclear | Yes | Yes | Yes | Low | Low | Low | Low |
| Shi *et al.*, 2022[34] | Yes | Yes | Yes | Low | Yes | Yes | Low | Yes | | Yes | No | High | Yes | Yes | Yes | No | Low | Low | Low | Low |
| Sun *et al.*, 2022[35] | Unclear | Yes | Unclear | High | Unclear | Yes | Low | Yes | | Yes | Yes | Low | Yes | Yes | Yes | Yes | Low | Low | Low | Low |
| Wang, C. *et al.*, 2022 [36] | Unclear | Yes | No | High | Unclear | No | High | Yes | | No | Unclear | High | Unclear | Yes | Yes | Yes | Low | Low | Low | Low |
| Wang, D. *et al.*, 2022[50] | Unclear | Yes | Yes | Low | Yes | Yes | Low | Yes | | Yes | No | High | Yes | Yes | Yes | Yes | Low | Low | Low | Low |

^*^ Patient selection, ^**^ Index test, ^***^ Reference standard. 1- Was a consecutive or random sample of patients enrolled? 2- Was a case-control design avoided? 3- Did the study avoid inappropriate exclusions? 4- Could the selection of patients have introduced bias? 5- Were the index test results interpreted without knowledge of the results of the reference standard? 6- If a threshold was used, was it prespecified? 7- Could the conduct or interpretation of the index test have introduced bias? 8- Is the reference standard likely to correctly classify the target condition? 9- Protocol specified for reference clinical evaluation 10- Were the reference standard results interpreted without knowledge of the results of the index test? 11- Could the reference standard, its conduct, or its interpretation have introduced bias? 12- Was there an appropriate interval between index tests and reference standard? 13- Did all patients receive a reference standard? 14- Did all patients receive the same reference standard? 15- Were all patients included in the analysis? 16- Could the patient flow have introduced bias? 17- Are there concerns that the included patients do not match the review question? 18- Are there concerns that the index test, its conduct, or its interpretation differ from the review question? 19- Are there concerns that the target condition as defined by the reference standard does not match the review question?
