# Supplementary figures and images for: The Diagnostic Accuracy of Metagenomic Next-Generation Sequencing in Diagnosing Pneumocystis Pneumonia: A Systemic Review and Meta-analysis
Source: Open Forum Infect Dis. 2023 Aug 18;10(9):ofad442. doi: 10.1093/ofid/ofad442 (PMC10478158; doi:10.1093/ofid/ofad442)

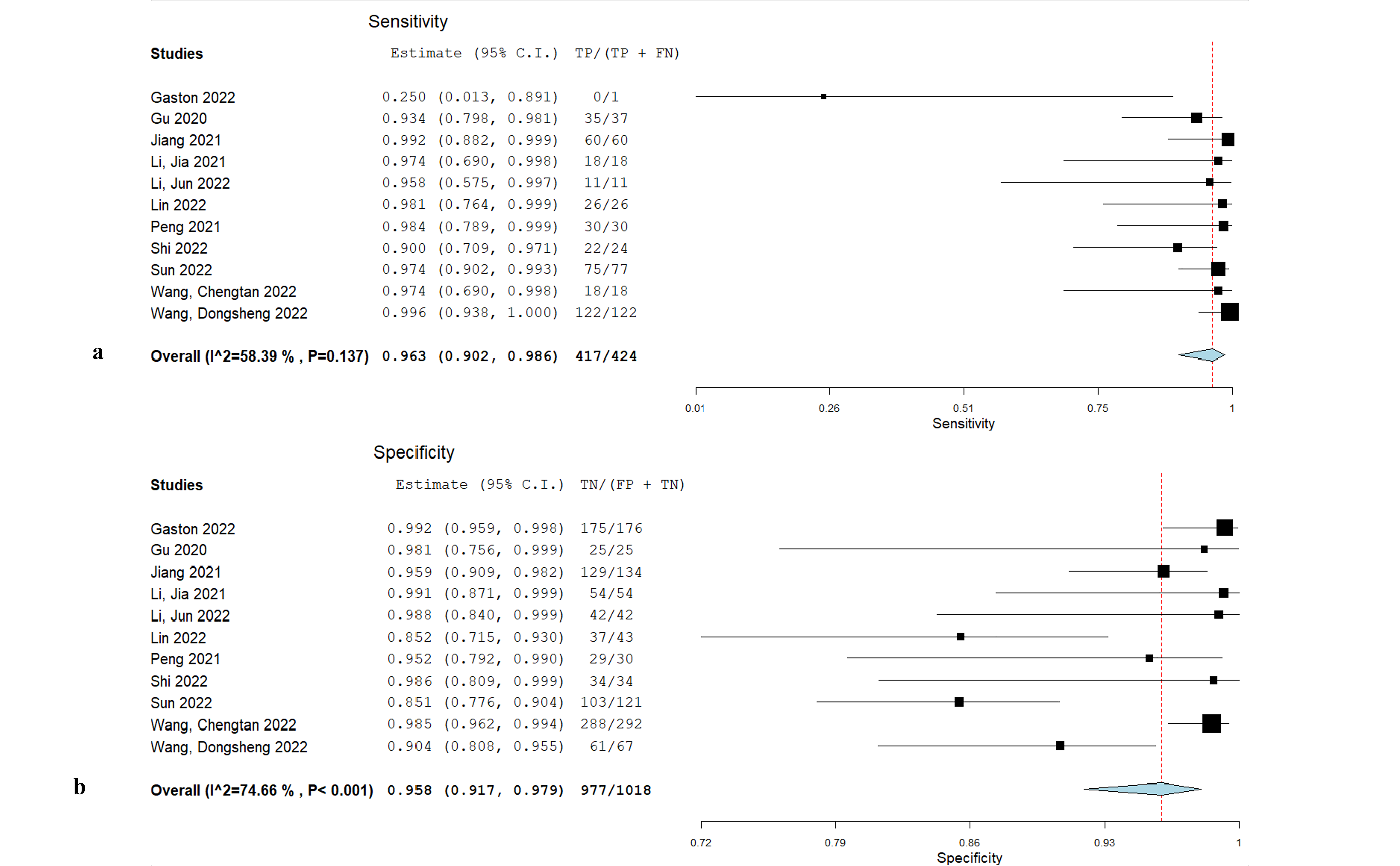

Supplement: ofad442_Supplementary_Data [file ofad442_supplementary_data.zip › Supp_fig_1_sens_spec.tif]
